# A review on *Lactococcus lactis*: from food to factory

**DOI:** 10.1186/s12934-017-0669-x

**Published:** 2017-04-04

**Authors:** Adelene Ai-Lian Song, Lionel L. A. In, Swee Hua Erin Lim, Raha Abdul Rahim

**Affiliations:** 1 Department of Microbiology, Faculty of Biotechnology & Biomolecular Sciences, University Putra Malaysia, 43400 Serdang, Selangor Malaysia; 2 Functional Food Research Group, Department of Biotechnology, Faculty of Applied Sciences, UCSI University, Kuala Lumpur, Malaysia; 3 Perdana University-Royal College of Surgeons in Ireland, Perdana University, Block B and D, MAEPS Building, MARDI Complex, Jalan MAEPS Perdana, 43400 Serdang, Selangor Malaysia; 4 Department of Cell & Molecular Biology, Faculty of Biotechnology & Biomolecular Sciences, University Putra Malaysia, Serdang, Selangor Malaysia

**Keywords:** *Lactococcus lactis*, Heterologous proteins, Recombinant proteins, Expression systems, Secretion, Surface display, Microbial cell factory

## Abstract

*Lactococcus lactis* has progressed a long way since its discovery and initial use in dairy product fermentation, to its present biotechnological applications in genetic engineering for the production of various recombinant proteins and metabolites that transcends the heterologous species barrier. Key desirable features of this gram-positive lactic acid non-colonizing gut bacteria include its generally recognized as safe (GRAS) status, probiotic properties, the absence of inclusion bodies and endotoxins, surface display and extracellular secretion technology, and a diverse selection of cloning and inducible expression vectors. This have made *L. lactis* a desirable and promising host on par with other well established model bacterial or yeast systems such as *Escherichia coli, Salmonella cerevisiae* and *Bacillus subtilis*. In this article, we review recent technological advancements, challenges, future prospects and current diversified examples on the use of *L. lactis* as a microbial cell factory. Additionally, we will also highlight latest medical-based applications involving whole-cell *L. lactis* as a live delivery vector for the administration of therapeutics against both communicable and non-communicable diseases.

## Background

Despite the common association of *Lactococcus lactis* with dairy products, the bacterium was originally isolated from plants where it was believed to be dormant, and only became active and multiplied in the gastrointestinal tract after being consumed by ruminants [1]. Originating from the streptococcus genus and re-classified into the *Lactococcus* genus in 1985, *L. lactis* is divided into three subspecies namely *L. lactis* subsp. *lactis*, *L. lactis* subsp. *cremoris*, and *L. lactis* subsp. *hordniae* [2]. Phenotypically, it is classified as a gram-positive, spherical, homolactate, non-sporulating, and facultative anaerobic gut bacteria with hundreds of strains and biovariants published to date [3, 4].

**Fig. 1:**
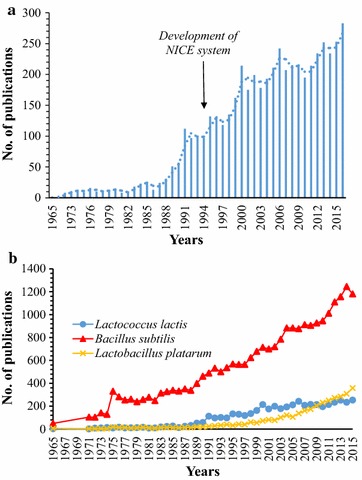
**a** *Graph* indicating an increasing trend of publications relating to *Lactococcus lactis* technological advancements and research. **b** Comparison of publications between Gram positive model organisms—*Bacillus subtilis, Lactobacillus plantarum* and *Lactococcus lactis* over the past 50 years

*Lactococcus lactis* has been used for centuries in the fermentation of food especially cheese, yoghurt, sauerkraut and the like, thereby rendering it’s generally recognized as safe (GRAS) status by the Food and Drug Administration (FDA). Apart from imparting flavour, *L. lactis* being a lactic acid bacteria (LAB) also produces acid which preserves food. Some strains further enhances this preservation property with the production of bacteriocins, thus reinforcing its role in the food industry. Other than its important function in food, *L. lactis* has become the model LAB when it comes to genetic engineering. Several factors including its small-sized fully sequenced genome (2.3 Mbp), and the development of successfully compatible genetic engineering tools such as cloning and expression systems with customizable options, have rendered it a desirable model. Over the past two decades, *L. lactis* has vastly extended its application from food to being a successful microbial cell factory (Fig. 1a), and on many occasions, acting as a gram-positive alternative to *Bacillus subtilis* and *Lactobacillus plantarum*, or its gram-negative counterpart, *Escherichia coli* (Fig. 1b) [5].

This review will cover the many aspects of *L. lactis* as a cell factory for an immense range of products as well as its role as a vehicle for delivery of therapeutics into the gastrointestinal system. It is to be used as an overview of the enormously extended biotechnological role that *L. lactis* has so far acquired, indicating other relevant and niche areas involving *L. lactis* where applicable.

## The lactococcal molecular toolbox

### Expression systems

One of the reasons *L. lactis* has emerged to become a successful microbial cell factory system is due to the wealth of genetic knowledge available spanning at least four fully sequenced lactococcal strains [6] and many existing expression systems. Various constitutive and inducible expression systems have been developed for *L. lactis* as screening of promoters using reporter genes such as beta-galactosidase are a commonly used strategy in developing novel lactococcal expression systems [7, 8]. P45 and P32 are commonly used as constitutive lactococcal promoters, but stronger promoters are still being discovered and developed to improve the system [9].

In most cases, inducible promoters are preferred over constitutive promoters as they provide better control to the user. There are various lactococcal inducible systems such as P_(Zn)*zitR*_ and Zirex system which are both regulated based on zinc availability where the former and latter are repressed and induced by the presence of zinc, respectively [10]. Other inducible promoters are PA170 which is induced by a decrease in pH during transition of culture to stationary phase thus making it autoinducible [11, 12] and P_*xylT*_ which is induced by xylose [13]. However, without doubt, the most successful lactococcal expression system to date is the nisin controlled gene expression (NICE) system developed by Kuipers and colleagues in 1995. Nisin is a 34-amino acid anti-microbial peptide whose biosynthesis is encoded by a cluster of 11 genes. Of the 11 genes, *nisR* and *nisK* regulate expression of the nisin genes. NisK is a histidine-protein kinase which resides in the cytoplasmic membrane and acts as a receptor for the nisin molecule. Upon reception of nisin, it activates *nisR* via phosphorylation, which in turn induces transcription of two promoters in the nisin gene cluster: PnisA and PnisF [14].

The host *L. lactis* NZ9000 is a derivative of the nisin-negative MG1363 strain with the *nisR* and *nisK* genes inserted into its chromosome [15]. When a gene of interest is placed downstream the PnisA promoter on a plasmid, expression of that gene can be induced by introduction of sub-inhibitory amounts of nisin (0.1–5.0 ng/ml). The most commonly used expression plasmid is pNZ8048 [15], which enables a gene insertion in the NcoI site overlapping the ATG start codon, allowing direct cloning of the gene fused to the *nisA* start codon [16]. Other commonly used strains and plasmids of the NICE system are reviewed in “10 years of NICE in *L. lactis*” [16]. Many of these NICE^®^ plasmids and compatible host strains developed by NIZO Food Research (Netherlands) are now commercially available, with many derivatives being established using NIZO systems including a system for TA-cloning, designated pNZ-T to facilitate restriction enzyme independent cloning [17].

### Secretion strategies

Secretion of heterologous proteins are mostly preferred compared to intracellularly expressed proteins due to advantages such as simpler purification steps, higher yields, and better target interactions [18]. In view of this, it is also advantageous to employ the secretion system when developing *L. lactis* as a host for heterologous protein production [19]. In addition, gram-positive bacteria have a monolayer cell wall that permits direct secretion into the extracellular environment in comparison to *E. coli* where secreted proteins are mostly stuck in the periplasm [20]. Furthermore, *L. lactis* only possesses a single extracellular housekeeping protease, HrtA, thereby reducing the chances of secreted heterologous proteins being degraded [21, 22].

Signal peptides (SPs) are N-terminal extensions of a protein which signals the host to target the protein towards the extracellular region by translocation across the cytoplasmic membrane and cell wall. While the sequence of SPs are vastly diversed, they display a common tripartite structure which includes the positively charged N-terminus, the hydrophobic H-region and the negatively charged cleavage region at the C-terminus [23]. In *L. lactis*, there is only one majorly secreted protein which is Usp45, whose function is still unknown [19]. Nevertheless, the native lactococcal Usp45 SP is the most successful SP used thus far for secretion in *L. lactis,* and was recently engineered through a series of mutations to further increase its secretion efficiency (SE) by 51% [24]. More recently, we have isolated a novel signal peptide, SPK1 from *Pediococcus pentasaceus*, with the ability to secrete heterologous proteins with efficiencies comparable to Usp45 in *L. lactis* [25]. When SPK1 was used to secrete β-cyclodextrin glucanotransferase, although secretion efficiency was higher than USP45, total yield was found to be lower [26], thus demonstrating the complex effects brought upon by SPs, not only on the secretion of heterologous proteins, but on total protein yield as well.

Apart from SPs, past literature have reported several other strategies which have proven to improve SE in *L. lactis* including the use of synthetic LEISSTCDA propeptide sequence (SPs are followed by a propeptide sequence which is cleaved after translocation to produce the mature protein) [23], and the use of a *hrtA* mutant strains (the only reported cell surface proteolytic housekeeping gene) [22]. In another strategy, it was shown that the secretion yield of some heterologous proteins can be improved in *L. lactis* when co-expressed with *B. subtilis* PrsA protein, which is a surface anchored protein with chaperon-like functions and have been shown to decrease degradation of exported proteins [27].

### Surface display systems

The thick and rigid cell wall of gram-positive bacteria as well as the lack of an outer membrane envelope has made them suitable for the cell surface display of proteins. Displaying proteins on bacterial cell wall allows the bacteria to act as carriers of proteins, especially antigens, and allow interaction of displayed proteins with targeted environments. There are five different types of protein anchors described in lactic acid bacteria; (1) transmembrane anchors: (2) lipoprotein anchors which binds to the cell membrane; (3) LPXTG-type cell wall anchoring domains; (4) AcmA-repeats anchor domain; (5) S-layer protein attachments which are bound to cell wall components [28].

In *L. lactis*, the most commonly used method for surface display of proteins is through the LPXTG sorting signal of surface-associated proteins which are recognized by the sortase enzyme, and covalently bound to the cell wall. In this method, the anchoring mechanism relies on the sortase activity as this membrane-anchored enzyme cleaves the sorting signal of the target protein at its pentapeptide motif (LPXTG) and promotes covalent anchoring of the target protein to the cell wall [29, 30]. However, non-covalent binding of cell surface proteins using lysin motifs (LysM) are also alternatively used, with the LysM of the autolysin AcmA being the most common [31]. More interestingly, non-covalent binding of antigens/proteins using AcmA has been shown to allow *trans* surface display, where proteins are displayed from the outside of *L. lactis* host cells, as we have previously shown [32]. Using this method, expression of heterologous proteins can be performed in a non-lactococcal host (e.g. *E. coli*), purified and bound non-covalently to the lactococcal cell wall simply by mixing the purified heterologous proteins to lactococcal cell cultures. More importantly, this enables the lactococcal cells to carry heterologous proteins without being genetically modified, a method which have also been demonstrated with Newcastle disease virus hemagglutinin-neuraminidase (HN) protein for specific targeting of breast cancer cells [33]. In addition, eukaryotic proteins which require post-translational modifications can also be expressed in eukaryotic hosts, and subsequently attached to *L. lactis* for delivery [34]. A variation of this method uses GEM (gram-positive enhancer matrix) particles which are killed non-recombinant lactococcal cells devoid of most intact cell wall components and intracellular materials. Antigens fused to streptococcal protein anchor enable them to be docked onto the peptidoglycan of GEM particles, which was also shown to elicit an immune response in nasally immunized mice [35]. A similar approach was also employed recently for subtilisin QK-2 using GEM [36]. The drawback of this system, however, is that the lactococcal cells are merely a carrier of the displayed protein, not a factory producing the proteins, thus repeated introduction of proteins displayed on lactococcal cells may be needed.

## *Lactococcus lactis* as a cell factory

### Production of industrial metabolites and enzymes

Naturally, *L. lactis* is a strictly homolactic fermentative bacteria which completely converts its carbon source into l-lactate from pyruvate through a very efficient lactate dehydrogenase (LDH) enzyme with a K_m_ value of 1.1 mM [37]. Lactic acid is an industrially important compound as it is used as an acidifier for preservation, as a flavour enhancing agent in the food industry [38], as an emulsifier and moisturizing agent in the cosmetic industry, and as an important raw material in the pharmaceutical industry [39]. Additionally, polymerization of lactic acid yields polylactic acid (PLA), which is a biodegradable thermoplastic polymer highly anticipated to potentially replace non-renewable oil based polymers [39]. While lactic acid remains the main product produced by *L. lactis* and other LAB, under different physiological conditions, three other enzymes apart from LDH also converts pyruvate: (i) α-acetolactate synthase (ALS) which is active at high pyruvate concentrations and low pH (≤6.0) [40]; (ii) pyruvate-formate-lyase (PFL) which is active under anaerobic conditions and at relatively high pH of >6.0 [41]; and (iii) pyruvate dehydrogenase (PDH) which is active under aerobic conditions and low pH (≤6.0) [42]. Therefore, *L. lactis* is also a natural factory for the production of many other aromatic acetylated products such as diacetyl, acetaldehyde and acetate, resulting from mixed fermentation. Nevertheless, LDH still dominates with maximal enzymatic activity at high sugar concentrations and high intracellular nicotinamide adenine dinucleotide dehydrogenase (NADH) levels [40, 43].

To date, metabolic engineering efforts in *L. lactis* have focussed primarily on customizing the prioritization of mixed fermentation products by re-routing lactate-pyruvate metabolism towards other industrially important products such as diacetyl, acetaldehyde and acetoin which are important flavour compounds in dairy products. This was achieved through the use of an LDH deficient *L. lactis* strain which consequently increased the amount of α-acetolactate in place of lactate, where the former is a reduced carboxylated form of diacetyl [40]. This together with other similar studies have indicated that LDH deficiency could result in >80% of lactose being converted into other fermentation products other than lactic acid through overproduction of ALS and activation of the diacetyl-acetoin pathway for pyruvate metabolism [44].

Another common metabolic engineering strategy in *L. lactis* involves manipulation of the NADH:NAD^+^ co-factor ratio which influences fermentation patterns because the in vivo activity of several central redox enzymes, namely glyceraldehyde 3-phosphate dehydrogenase (GADPH), PDH, LDH, alcohol dehydrogenase (ADH) and NADPH oxidase (NOX), are significantly influenced by this ratio. For example, the *nox* gene which encodes NADH oxidase converts molecular oxygen to water at the expense of NADH. Overexpression of NOX diminishes the NADH pool, and increases NAD^+^, thereby re-routing pyruvate from the NADH-dependent LDH pathway to either the NADH independent ALS pathway or the NAD^+^ dependent PDH pathway. This strategy has been shown to be successful in shifting homolactic fermentation to mixed-acid fermentation with acetate and acetoin as main products, while producing α-acetolactate and diacetyl in small amounts [45]. Combining this with disruption of the gene encoding α-acetolactate decarboxylase also yielded high diacetyl production from glucose and lactose [46]. In fact, it was shown that the adjustment of aeration levels alone, even in minute amounts without any metabolic engineering was able to greatly re-route up to 80% of fermentation products from lactate to other products such as formate, acetate, and ethanol [47]. On a different note, *L. lactis* has also been engineered to be a factory for the production of sweeteners, including the introduction of heterologous pathways or enzymes such as alanine dehydrogenase from *Bacillus sphaericus* for the production of l-alanine [48].

More recently, the emphasis of metabolic engineering in *L. lactis* have somewhat shifted towards increasing the production of non-food flavouring metabolites. Examples include the B vitamins, primarily folate (B11) and riboflavin (B2), which were overexpressed in *L. lactis* using the NICE system [49–51]. These reports highlight *L. lactis* as a food-grade platform where the production of multivitamins from guanosine triphosphate (GTP) precursors can be increased by 3 to 10 folds following overexpression of a GTP biosynthetic enzyme (GTP cyclohydrolase I) [49, 50].

Other recent studies have shown the potential of *L. lactis* in bacteriocin production as a bio-preservative against *Listeria monocytogenes* [52, 53] and these bacteriocins have been found useful also for clinical applications [54] via prevention/reduction of biofilm formation. LAB bacteriocins are antimicrobial peptides which have been ribosomally synthesized at transcriptional and post-transcriptional levels; this confers auto-immunity to the producer strain [55]. Examples of more recent bacteriocins from *L. lactis* include lacticin 3147 [56], lacticin Q/Z [57] and LsbB [58]. However, the most well-known and best characterised lantibiotic is nisin (term “lantibiotic” derived from Schnell [59] as lanthionine containing antibiotic), which had been discussed in depth in the preceding section. Current efforts are ongoing [60–62] to characterise bacteriocins from *L. lactis* and some favourable attributes for applications include acid stability and thermotolerance to high temperatures in addition to improvement in production systems.

On another note, *L. lactis* has also been engineered to produce ethanol as biofuels when supplemented with cheap renewable feedstock waste products [63]. A summary of industrial products produced on a lactococcal platform is summarized in Table 1.

**Table 1:** List of industrial enzymes and compounds produced from various *Lactococcus* *lactis* strains

Industrial type & products	Applications/functions	*Lactococcus lactis* strain	References
Compounds			
Lactic acid	Preservative, flavouring, polylactic acid, plastic, emulsifier, moisturizer	All strains	[43]
Acetoin/diacetyl	Flavouring	CRL264	[44]
l-alanine	Sweetener	AlaDH^+^LDH^−^	[48]
Linalool	Flavouring	NZ9000	[64]
Germacrene D	Antimicrobial, insecticidal, pheromones	NZ9000	[65]
β-Sesquiphellandrene	Antimicrobial, antioxidant, anticancer	NZ9000	[66, 67]
Hyaluronic acid	Cosmetics, medical	NZ9020	
Vitamins			
Folate (B11)	Health supplements	NZ9000	[49–51]
Riboflavin (B12)	Health supplements	NZ9000	
Biofuels			
Ethanol	Energy source	CS4435	[63]
Peptides			
Bacteriocin	Anti-microbial, preservative	NZ9000	[52, 68]
Brazzein	Sweetener	*N/S*	[69]
Mabinlin II	Sweetener	*N/S*	[70]
Nisin Z	Food preservative	F44	[71]
Enzymes			
β-Cyclodextrin glucanotransferase	Starch degradation	NZ9000	[26]
Coumarate CoA ligase (4CL)	Metabolic engineering	FI9974	[72]
Alcohol acyltransferase (SAAT)	Metabolic engineering	NZ9000	[64]
Linalool/nerolidol synthase (FaNES)	Metabolic engineering	NZ9000	[64]
Sesquiterpene synthase	Metabolic engineering	NZ9000	[65]
3-Hydroxy-3-methylglutaryl CoA reductase (HMGR)	Metabolic engineering	NZ9000	[66]
Bile salt hydrolase (BSH)	Intestinal metabolism, probiotics	NZ3900	[73]
Acid urease	Urea hydrolysis	*N/S*	[74]

*N/S* not specified

### Production of therapeutics

Due to its immunomodulatory properties and its ability to survive passage through the gastrointestinal tract (GIT), yet not colonize the gut unlike *Lactobacillus* spp., *L. lactis* has been used as a vehicle to deliver therapeutics such as cytokines into the human body. The first evidence of such applications was published in Steidler et al. [75], where engineered secretion of interleukin-10 (IL-10) in *L. lactis* was used to treat inflammatory bowel disease (IBD) in colitis-induced mice. Since then, *L. lactis* secreting IL-10 has gone into clinical trials and concurrently ushered in the emergence of a genetically modified thymidine auxotrophic *L. lactis* strain for biological containment which disallows growth of the bacteria unless provided externally with thymidine or thymine [76, 77]. While clinical trial results were not as promising as hoped, this bio-containment strategy was highly successful, making it a safe genetically modified organism (GMO) strain which addresses concerns relating to release to the public. Since the use of IL-10 for IBD treatment, many other therapeutics have been produced in *L. lactis* (Table 2) for the treatment of IBD including other cytokines, antioxidant enzymes and protease inhibitors [78].

**Table 2:** Recombinant therapeutics produced from various *Lactococcus lactis* strains

Therapeutic type & products	Disorder/disease	*Lactococcus lactis* strain	References
Cytokines/ligands			
Interleukin-6 (IL-6)	Adjuvant	IL1403	[123]
Interleukin-10 (IL-10)	Adjuvant, hypersensitivity type I, inflammatory bowel disease (IBD)	*N/S*	[75, 79]
Interleukin-12 (IL-12)	Adjuvant; hypersensitivity type I; asthma	NZ9000	[75, 79, 81]
Interleukin-18 (IL-18)	Adjuvant, immunomodulatory,	MG1363	[124]
Hemagglutinin-neuraminidase (HN) protein of NDV	Breast cancer	NZ9000	[33]
RANKL	Cancer vaccine adjuvant	IL1403	[84]
Transforming growth factor beta 1 (TGF-β1)	IBD	NZ9000	[82]
Epidermal growth factor (EGF) Trefoil factor 3 (TFF3)	Wound healing	NZ9000	[125]
Kisspeptin (KiSS 1)	Colorectal cancer	NZ9000	[88]
Insulin-like growth factor I (IGF-I)	Colitis	NZ9000	[83]
Allergens			
Peanut allergen (Ara 2)	Hypersensitivity type I	CHW9	[85]
Birch allergen (Bet v1)	Hypersensitivity type I	NZ9800	[86]
House dust mite allergen (Der p2)	Hypersensitivity type I	NZ9000	[87]
Enzymes			
Subtilisin QK-2	Thrombosis	NZ9000 & NZ3900	[36]
Heme oxygenase-1 (rmHO-1)	Acute colitis	NZ9000	[126]
Vaccines/antigens			
Tetanus toxin fragment C (TTFC)	Tetanus	UCP1054	[96, 97]
HPV-16-E7	HPV-16 induced cancers	NZ9000	[101, 120]
Pneumoccal antigen	Pneumococcal infections, meningitis	*N/S*	[35, 104]
Listeriolysin O & mt Internalin A	Listeriosis	NZ9000	[34]
Glycosylated tyrosinase related protein-2 (TRP-2)	Skin cancer	MG1363	[102]
Carcinoembryonic antigen (CEA)	Colon cancer	NZ9000	[127]
*Plasmodium falciparum* recombinant antigen (R0.10C)	Malaria	*N/S*	[105]
Influenza virus nucleoprotein (NP)	Influenza	NZ9000	[128]
*Shigella* IpaB and IpaD	Shigellosis	PA1001	[106–108]
Neuraminidase (NA1)	Avian influenza H5N1	NZ3000	[109, 110]
Hemagglutinin (HA1)	Avian influenza H5N1	NZ9000	[111]
Hemagglutinin (HA1)	Avian influenza H1N1	NZ9000	[112]
M2e antigen	Avian influenza H5N2	LM2301	[113]
IBV multi-epitope gene*EpiC*	Avian bronchitis	NZ3900	[114]
Campylobacter rCjaAD antigen	Avian gastroenteritis	IL1403	[115]
GroEL, heat-shock protein	Brucelosis	NZ9000	[116]
Cu–Zn SOD of *Brucella abortus*	Brucelosis	NZ9000	[129]
Mycobacterial ESAT-6 antigen	Tubercolosis	*N/S*	[117]
D1 and D4 aerolysin	*Aeromonas* spp. infection	Lac-D1ae	[118]
SiMA antigen	*Streptococcal* infection	BFE920	
Myelin epitopes	Multiple sclerosis, encephalomyelitis	IBB360	[130]
T1D autoantigens	Type-1 diabetes mellitus	*N/S*	[131]
Enterohemorrhagic *Escherichia coli* (EHEC) antigen (EspB)	EHEC infection	*N/S*	[132]
Multi-urease epitopes (CTB-UE)	*Helicobacter pylori* infection	NZ9000	[133]
Helicobacter pylori hspA	*Helicobacter pylori* infection	NZ3900	[71]
HIV-1 Gag-p24	Human immunodeficiency virus (HIV) infection	*N/S*	[134]
Capsid protein of porcine circovirus type 2 (PCV2)	Swine circovirus associated disease	*N/S*	[135]
*Staphylococcus aureus* HtrA protease	*Staphylococcal* infection	IL1403	[136]
*Staphylococcus aureus* clumping factor A (ClfA)	*Staphylococcal* infection	*N/S*	[137]
Hepatitis E virus antigen	Hepatitis E virus infection	NZ3900	[138]
Toxin A/B (TcdA/B)	*Clostridium difficile* infection	*N/S*	[139]
F and G glycoproteins of Respiratory syncytial virus	Upper respiratory tract infection	NZ9000	[140]
Others			
HSP65-6IA2P2	Type 1 diabetes mellitus	NZ9000	[90]
Gamma-amino butyric acid (GABA)	Hypotensive, anti-cancer, anti-anxiety	All ssp. Lactis	[93]
*Bacillus thuringiensis* crystal protein Cry5B	Anthelminthic	NCK203	[89]
Serine protease inhibitors	IBD	NZ9000	[82]
Glucagon like peptide-1 (GLP-1)	Type 2 diabetes mellitus	*N/S*	[141]

*N/S* not specified

When it comes to hypersensitivity, IL-10 secreting *L. lactis* strains have also been investigated as treatment against food allergy such as cow’s milk allergy [79]. In this study involving β-lactoglobulin-induced anaphylaxis in mice, it was shown that oral administration of a recombinant *L. lactis* delivering IL-10 gastrointestinally prior to sensitization was able to induce immunotolerance towards the allergen, thus reducing food-induced anaphylaxis. Recombinant *L. lactis* producing IL-12, a T-helper 1 (Th1) bias cytokine has also been investigated for the treatment of asthma, successfully skewing the Th2 dominant immunologic response in murine models of asthma to a Th1 response which simultaneously elevates interferon gamma (IFN-γ) whilst reducing IL-4 levels [80]. To date, *L. lactis* has been used to co-produce or secrete a wide range of other adjuvants and growth factors. Successful examples include murine IL-12 [81], transforming growth factor beta 1 (TGF-β1) [82], insulin-like growth factor I [83], receptor activator of nuclear factor kappa-B ligand (RANKL) [84] and others as detailed in Table 2.

Apart from the use of *L. lactis* in delivering cytokines to alleviate allergy symptoms, *L. lactis* has also been developed as factory for production and purification of the allergen itself. In 2007, Glenting et al. reported the production of immunologically active recombinant peanut allergen Ara 2 in *L. lactis* with high yields [85]. Recombinant allergens are arguably superior over natural allergen owing to its purity and batch to batch consistency. Furthermore, in addition to playing the role of factory in producing allergens, *L. lactis* can simultaneously be used to deliver allergens such as the major birch allergen Bet-v1 [86], and the house dust mite (HDM) allergen Der p2 [87] through the GIT to achieve immunotolerance prior to sensitization. *L. lactis* are naturally great delivery vehicles for allergy immunotherapy as many non-recombinant LAB by itself, including *L. lactis,* have shown anti-allergic effects through their immunomodulatory effects, owing to their cell wall components and other non-established factors.

Recently, in the field of anti-cancer therapeutics, recombinant *L. lactis* NZ9000 was used to secrete tumour metastasis-inhibiting peptides such as KiSS1 which inhibited HT-29 cell proliferation and migration through the induction of apoptosis pathways and by down regulating matrix metallopeptidase 9 (MMP-9) expression. This suggested a possible role for *L. lactis* as a cell factory for colorectal cancer therapeutics [88]. Other examples of therapeutics produced using *L. lactis* as a microbial cell factory include subtilisin QK-2 as an anti-thrombotic agent [36], BT crystal protein Cry5B as an anthelminthic [89], heat shock protein (hsp) 65-6IA2P2 against type 1 diabetes [90] and many others as summarized in Table 2.

In addition to protein- and whole cell-based therapeutics, metabolites with medicinal applications are also produced by *L. lactis.* An example is γ-amino butyric acid (GABA), which is a non-protein amino acid with hypotensive, anti-cancer, anti-anxiety and diuretic properties [91, 92]. Naturally produced GABA are generally favourable compared to its chemically synthesized counterpart, and also contributes to pH tolerance, hence making LAB excellent GABA producing candidates [93]. In *L. lactis*, GABA production can also be used to differentiate between *L. lactis* ssp. *lactis* and *L. lactis* ssp. *cremoris* as the former produces GABA while the latter does not [94]. Other examples of medicinal metabolites successfully synthesized by *L. lactis* includes hyaluronic acid, which is a carbohydrate polymer used in wound healing, dermatitis and cosmetic-based applications [67].

### Vaccine delivery system

Without doubt, one of the most exciting aspects of modern *L. lactis* usage is as a factory for antigen production, thus allowing the bacteria to act as live vaccines. Using LAB as vaccine carriers is appealing as they are able to induce both mucosal and systemic immune responses, have adjuvant properties, and is free from risks associated with the use of conventional attenuated live pathogens such as *Salmonella* spp. and *Mycobacterium* spp. [95]. When it comes to vaccine design, the capability of *L. lactis* to surface display antigens also transforms it into the preferred host with increased immunogenicity compared to its intracellularly expressed or secreted counterparts [96]. One of the earliest pioneering vaccine initiatives using *L. lactis* involved expressing tetanus fragment toxin C (TFTC), which was highly successful in eliciting immune responses in mice, especially when administered together with IL-2 and IL-6 adjuvants [97]. Since then, a variety of antigens against both human and animal diseases have been expressed, secreted and surface displayed in *L. lactis* as detailed in several past reviews with a comprehensive list of LAB-based vaccines [98–100], together with an updated list as detailed in Table 2.

Over the past decade, the emergence of cancer vaccines developed via a lactococcal platform has also been gaining momentum following the onset of prokaryotic antigen production. These include a vaccine against human papilloma virus type-16 induced tumours where *L. lactis* surface displaying the E7 antigen whist secreting IL-12 was shown to provide full prophylactic protection in immunized mice and was also able to induce regression of palpable tumours in tumour-induced mice [101]. Other cancer antigens expressed using *L. lactis* includes glycosylated tyrosinase related protein-2 (TRP-2) tumour antigen against melanoma (although this has not gone to animal trials) [34] and carcinoembryonic antigen (CEA) against colon cancer in mice [102]. The latter showed successful induction of immune response in mice as indicated by higher levels of CEA-specific secretory IgA compared to controls.

Being capable of heterologous protein expression, characterization of bacterial and viral virulence factors using *L. lactis* was also made possible without the pathogen’s clinical manifestations. A very recent example is the expressive characterization of the *Streptococcus mutans* surface glycoprotein, Cnm in *L. lactis* which was found to mediate binding to extracellular matrix (ECM) proteins in a rabbit model of infective endocarditis [103]. In addition, virulence factors comprising mutated internalin A and listeriolysin O (LLO) from food-borne pathogen *L. monocytogenes* have been proposed for use in DNA vaccination using *L. lactis* as hosts for plasmid production [104]. Recombinant *L. lactis* strains harbouring viral antigens such as influenza A nucleoprotein (NP) have also been studied, and shown to elicit superior immunogenicity, especially when coupled with oral adjuvants such as cholera toxin B (CTB) subunits [105].

Various lactococcal-based vaccines for animal diseases have also been developed, mostly with favourable results. In poultry diseases, extensive research has been performed against the H5N1 virus, using *L. lactis* as a vaccine delivery system via oral and intranasal administration routes in chickens and ferrets [106–109]. It was demonstrated that these lactococcal vaccines were able to induce high hemagglutinin A (HA)-specific serum IgG and fecal IgA, with the secreted form being more efficient than the intracellularly expressed vaccine [109]. Following this, surface display of HA antigen onto *L. lactis* surface using PgsA anchor motif administered orally together with (CTB) as adjuvant was also found to elicit high antigen-specific cell-mediated responses in mice when challenged with lethal dosages of H5N1 [110]. This demonstrated the stability and immunogenicity of surface anchored proteins as per many previous studies. Other lactococcal based vaccines developed or under development for the poultry industry include those against H1N1 [111], H5N2 [112], avian infectious bronchitis virus [113] and infections by *Campylobacter jejuni* [114].

One of the earliest uses of *L. lactis* in the livestock industry was reported a decade ago, where GroEL heat shock protein from *Brucella abortus* was expressed and secreted as a vaccine candidate. However, its intracellular expression was shown to be unstable with a low secretion efficiency [115]. Through technological advancements in expression and secretion systems, consecutive attempts were proven more successful when oral administration of recombinant lactococcal strains secreting Cu–Zn superoxide dismutase (SOD) of *B. abortus* was found to render protective immunity against brucellosis when tested in mice [116]. Very recently, oral administration of recombinant insulin-like growth factor I (IGF-I) expressed in *L. lactis* also reported good biological activity, where symptoms and development of dextran sodium sulphate (DSS)-induced colitis in mice were attenuated [83]. Use of *L. lactis* has also made hallmarks in the aquaculture industry where lactococcal-based vaccines against *Aeromonas hydrophila* using D1 and D4 aerolysin genes were developed with increased survival in tilapia fish when administered intraperitoneally and orally [117]. Lactococcal expression of the SiMA antigen, a *Streptococcus iniae* membrane protein, has also incurred significant vaccinative and probiotic effects in olive flounders [118].

At present, enhancements to the lactococcal vaccine delivery system are continuously being carried out, amongst which, includes the recent incorporation of the cell-surface anchored fibronectin binding protein A (FnBPA) from *Staphylococcus aureus* which functions to increase immunomodulatory properties of *L. lactis* strains during mucosal delivery as a live DNA vaccine vector [119, 120]. *L. lactis* shuttle vectors such as the pNZ:vig [121] and pPERDBY reporter plasmid [122] for the delivery of DNA vaccines to mammalian cells have also been developed and in the latter shown to perform efficiently in the absence of invasive proteins or relevant chemical treatments. As an effort to provide protection against gastric digestion, enteric coated encapsulation of lactococcal vaccines have also been explored with superior levels of antibodies being elicited, conferring full protection against H5N1 in mice [109]. It is apparent from Table 2 that the use of *L. lactis* as a factory for antigens and adjuvants renders it a very promising live bacterial vaccine host, consequently turning it into one of the most extensively researched areas.

### Production of heterologous plant-based proteins

Although unconventional, *L. lactis* has also been engineered as a cell factory for the production of both plant proteins and bioactive compounds as described in Table 1. Coumarate CoA ligase (4CL) from *Arabidopsis thaliana* was the first functional plant protein to be expressed in *L. lactis* [72]. A year later, brazzein, a sweet tasting plant protein, extracted from the fruit of the West African plant, *Pentadiplandra brazzeana,* was successfully expressed, albeit in low amounts [142]. The establishment of plant protein expression in *L. lactis* soon led to the metabolic engineering and consequent production of industrially applicable secondary metabolites. In 2007, alcohol acyltransferase (SAAT) and linalool/nerolidol synthase (FaNES) of strawberry were reportedly expressed in *L. lactis,* leading to the production of the flavouring and scent compound, linalool [64], which is used in various essential oil-containing cosmetics and fragrances.

Our research group has also successfully expressed two plant terpene synthases from orchid [65] and kesum (*Persicaria minor*) [66] in *L. lactis* leading to the production of germacrene D and β-sesquiphellandrene, respectively. Interestingly, *L. lactis* uses the mevalonate pathway (MVA) for terpenoid biosynthesis, a pathway more commonly found in eukaryotes. Most prokaryotes such as *E. coli* uses another terpenoid biosynthesis pathway called the 2-C-methyl-d-erythritol-4-phosphate (MEP) pathway, hence almost all metabolic engineering research for heterologous plant terpenoid production have been focussed on the MEP pathway of *E. coli* or the MVA pathway of yeast. At present, the lactococcal MVA accommodates much room for optimization as initial attempts to metabolically engineer this pathway resulted in more than doubling of sesquiterpenes produced [143].

### Production of membrane-based proteins

Membrane proteins are typically difficult proteins to express and purify due to their nature which includes low abundance, relatively high hydrophobicity, instability and various topologies including polytopic proteins with multiple transmembrane regions. Due to these limitations, only about 400 three-dimensional membrane protein structures have been elucidated compared to 40,000 soluble proteins, which accounts for a mere 0.01% [144]. Over the past two decades, *L. lactis* has been proven to be an excellent host for the expression of membrane proteins due to several advantages: (i) they are amino acid auxotrophs allowing incorporation of labels for detection, (ii) they only have a single membrane layer compared to *E. coli*, (iii) they have a small genome size with little proteolytic activity, and (iv) they come with extensive genetic engineering tools including the highly efficient and well tested NICE system.

To date, there are close to 100 membrane proteins expressed in *L. lactis* using the NICE system alone, including both prokaryotic and eukaryotic membrane proteins [144]. Kunji and colleagues [145] were the pioneers in using *L. lactis* as an alternative host for membrane protein overexpression of eukaryotic expression, successfully expressing human Lys–Asp–Glu–Leu KDEL receptor and mitochondrial carriers from yeasts and fungi. Prior to this, only prokaryotic membrane protein expressions were performed in *L. lactis*, mostly with homologous proteins, some which were able to reach up to 30% of total membrane proteins [144]. Most recently, *L. lactis* was successfully used to express rat and human membrane proteins involved in liver detoxification with higher yields than conventional *E. coli* and *Saccharomyces cerevisiae* expression systems [146].

*Lactococcus lactis* has also been developed as an alternative system for the production of plant membrane proteins, using *A. thaliana* peripheral and intrinsic proteins as a model [147]. A lactococcal cloning strategy compatible to Gateway entry vectors were established where available Gateway-based *A. thaliana* cDNA libraries were cloned into Gateway entry vectors and transferred into a destination vector (pBS-RFA) through recombination, thus allowing for proper reading frame preservation. The gene of interest is then excised and cloned into pNZ8148 and expressed as usual using the NICE system. This method allows for the use of Gateway available cDNA libraries, which in essence, cannot be used with the lactococcal NICE system due to host incompatibility. Using this method, six *A. thaliana* membrane proteins were produced of which three were successfully solubilized and purified with two of them being shown to be functional [147].

Using modified Gateway-compatible systems, research efforts were extended to the expression of 20 different membrane proteins from plants, human and bacteria in six different hosts including *L. lactis*, where Lactococcal-based expression was found to be an efficient and valuable alternative to *E. coli*, many times complementing proteins which were unsuccessfully produced in the latter [148]. While *E. coli* remains the superior host in terms of production yield in most cases, the fusion of proteins with Mistic, a 13 kDa protein from *B. subtilis* was reported to facilitate and improve membrane protein production in *L. lactis*. A more recent research validated the use of Mistic in successfully boosting the expression of both eukaryotic and prokaryotic membrane protein expression in *L. lactis* [149].

## Challenges and future prospects

While manipulations involving *L. lactis* enables various heterologous genes to be expressed, its yield is very much case-dependant, with lesser problems when it comes to closely related organisms such as *Streptococcus* spp.*, Enterococcus* spp.*, Staphylococcus* spp. and low-GC *Lactobacillus* spp. However, the greatest obstacle still revolves around its codon usage and/or distribution of rarely used codons [16, 150]. Additionally, gram protein per litre secretion in the microaerophilic *Lactococcus* spp. is still generally less robust when compared to the aerobically growing *B. subtilis*. Previously, genes encoding toxic gene products typically resulted in unsuccessful cloning attempts, further challenging the already-low transformation rate, but this was overcome by incorporating the nisA promoter in single copy on the chromosome [151, 152].

Following up on the use of NICE system, controlling the consistency of dosages and delivery of therapeutic molecules is also difficult to predict with *L. lactis* owing to the loosely controlled stability and small intestinal absorption of nisin, which ultimately influences the pharmacokinetics and pharmacodynamics interplay [69]. Another challenge involves the fine balance between switching from homolactic to mixed-acid fermentation in *L. lactis* which remains unresolved to-date [153], and elucidating this may vastly improve the potential of *L. lactis* as a cell factory.

In spite of limited systematic studies available on the acquired antibiotic resistance especially from food due to *L. lactis* designated GRAS status, a study documenting multiple drug efflux proteins attributing resistance to ethidium bromide was discovered in *L. lactis* subsp. *lactis* MG1363 [154]. Since much of the use for *L. lactis* as a cell factory involves ingestion or uptake into the host, there lies a risk for horizontal transfer of these efflux pumps to other commensal or potentially pathogenic bacteria. In other words, transit of *L. lactis* through the GIT which is frequently exposed to antibiotics may cause susceptible gene exchange with the surrounding flora, potentially leading to antibiotic resistant strains. In addition, release of chloramphenicol resistant pNZ-harbouring *L. lactis* strains into the environment is also a grave concern.

Therefore, it is imperative that guidelines mandating the management of *L. lactis* as a cell factory be put in place, especially for new strains, that conform to pre-marketing safety profiling and post-marketing follow-up to determine their acceptability [155]. Many lactococcal system developments have also incorporated food-grade markers in place of antibiotic resistance markers so as to maintain the GRAS status of *L. lactis* [156]. Alternatively, much consideration should be given on privileged aspects whereby recombinant *L. lactis* should be used. Examples include basis of delivered molecule, persistence of strain, robustness of the expression system, composition of different molecule subtypes and delivery to specific eukaryotic cells [157]. While its long history and safe use may be somewhat arguable, oftentimes, modifications to the final engineered product may bring about unexpected consequences [158], which also explains why only a few cell factories involving *L. lactis* have actually entered human trials.

When it comes to cancer vaccines, new knowledge and advancements in immunomodulation and microencapsulation technology of live lactococcal delivery vectors coupled with the richness of gut-associated lymphoid tissues (GALTs) have consequently opened up a gateway in exploiting future vaccination efforts via an oral-mucosal route, where we predict future research efforts being streamlined towards the lactococcal-based production of recombinant tumour antigens, especially against gastrointestinal malignancies over the next decade. In essence, this approach slingshots the practical use of peptide-based vaccines by overcoming its existing shortcomings such as its poor plasma stability and systemic half-life when administered in vivo.

## Conclusions

*Lactococcus lactis* have come a long way from being a food bacterium to a microbial cell factory for the production of industrially important products with potentially great bio-economic value, especially in the medical field. In spite of its limitations, there is still much room for improvement of the lactococcal system as a microbial cell factory since its molecular toolbox is still relatively limited compared to those available for *E. coli*. An expansion of said toolbox would be akin to opening a Pandora’s box, thus allowing further potential especially in terms of genetic and metabolic engineering to overcome limitations highlighted above.

## Abbreviations

MEP: 2-C-methyl-d-erythritol-4-phosphate
4CL: 4-coumarate-CoA ligase
ADH: alcohol dehydrogenase
ALS: alpha-acetolactate synthase
CEA: carcinoembryonic antigen
CTB: cholera toxin B
DSS: dextran sulfate sodium
EHEC: enterohemorrhagic *Escherichia coli*
ECM: extracellular matrix
FnbpA: fibronectin-binding protein A
FDA: Food and Drug Administration
GABA: gamma-amino butyric acid
GIT: gastrointestinal tract
GMO: genetically modified organism
GEM: gram-positive extracellular matrix
GRAS: generally regarded as safe
GAPDH: glyceraldehyde 3-phosphate dehydrogenase
GTP: guanosine triphosphate
GALT: gut-associated lymphoid tissue
Hsp: heat shock protein
HA: hemagglutinin
HN: hemagglutinin-neuraminidase
HIV: human immunodeficiency virus
IBV: infectious bronchitis virus
IBD: inflammatory bowel disease
IGF-I: insulin-like growth factor I
IFN: interferon
IL: interleukin
KDEL: Lys–Asp–Glu–Leu
KRAS: Kirsten rat sarcoma viral oncogene homolog
LDH: lactate dehydrogenase
LAB: lactic acid bacteria
LLO: listeriolysin O
LysM: lysine motif
MVA: mevalonate pathway
NOX: NADPH oxidase
NDV: Newcastle disease virus
NADH: nicotinamide adenine dinucleotide dehydrogenase
NICE: nisin-controlled gene expression
NP: nucleoprotein
PLA: polylactic acid
PCV: porcine circovirus
PDH: pyruvate dehydrogenase
PFL: pyruvate-formate-lyase
SE: secretion efficiency
SP: signal peptides
SOD: superoxide dismutase
Th: T-helper
TGF-β1: transforming growth factor beta 1
T1D: type-1 diabetes
