# Erratum to: A review on *Lactococcus lactis*: from food to factory

**DOI:** 10.1186/s12934-017-0754-1

**Published:** 2017-08-09

**Authors:** Adelene Ai‑Lian Song, Lionel L. A. In, Swee Hua Erin Lim, Raha Abdul Rahim

**Affiliations:** 1 Department of Microbiology, Faculty of Biotechnology & Biomolecular Sciences, University Putra Malaysia, 43400 Serdang, Selangor Malaysia; 2 Functional Food Research Group, Department of Biotechnology, Faculty of Applied Sciences, UCSI University, Kuala Lumpur, Malaysia; 3 Perdana University-Royal College of Surgeons in Ireland, Perdana University, Block B and D, MAEPS Building, MARDI Complex, Jalan MAEPS Perdana, 43400 Serdang, Selangor Malaysia; 4 Department of Cell & Molecular Biology, Faculty of Biotechnology & Biomolecular Sciences, University Putra Malaysia, Serdang, Selangor Malaysia

## Erratum to: Microb Cell Fact (2017) 16:55 DOI 10.1186/s12934-017-0669-x

Following the publication of this article [1], it was brought to our attention that unfortunately there is an error in the Abstract.

The Abstract currently reads: “…This have made *L. lactis* a desirable and promising host on par with other well established model bacterial or yeast systems such as *Escherichia coli*,* Salmonella cerevisiae* and *Bacillus subtilis*…”.

This should instead read: “…This have made *L. lactis* a desirable and promising host on par with other well established model bacterial or yeast systems such as *Escherichia coli*,* Saccharomyces cerevisiae* and *Bacillus subtilis*…”.
